# INCREASING PHYSICAL ACTIVITY POST-KIDNEY TRANSPLANT: A PILOT RANDOMIZED CONTROLLED TRIAL

**DOI:** 10.1093/geroni/igz038.1919

**Published:** 2019-11-08

**Authors:** Tara O’Brien, Cynthia Russell, Donna Hathaway

**Affiliations:** 1 The Ohio State University, Columbus, United States; 2 University of Missouri-Kansas City, Missouri-Kansas City, United States; 3 University of Tennessee Health Science Center, Memphis, Tennessee, United States

## Abstract

Older kidney transplant recipients are at risk for graft failure and death due to lack of physical activity. Physical activity after transplant is the most modifiable non-pharmacological factor for improving physical function. One personal system intervention called, SystemCHANGE™ in combination with activity trackers, holds promise for increasing physical activity among this population. The purpose of this pilot randomized controlled trial was to evaluate the efficacy of SystemCHANGE™ on increasing average daily steps in older (age 60 and over) kidney transplant recipients from baseline to 6 months. The intervention group met monthly to implement a successful personal system solution based on their daily routines and step-data collected from the activity tracker. The control group received monthly educational information on healthy living with a transplant. Participants were randomized 1:1 to the intervention or control group. The sample consisted of 31 participants (n = 15 intervention, and n = 16 control). No significant differences were found at baseline among the groups for demographics, self-efficacy and health outcomes (blood pressure, weight, waist circumference, 6 minute Walk Test). However, the intervention group had greater increase in the average daily steps from baseline to 6 months (mean ± SD: 1511 ± 2320) as compared to the control group (181 ± 2419). The between-group difference was of medium effect size (d = .56).The data suggests SystemCHANGE™ in combination with activity trackers may be feasible for older kidney transplant recipients to enhance daily steps.

